# Supplementary material for: Rapid detection of social interactions is the result of domain general attentional processes
Source: PLoS One. 2022 Jan 14;17(1):e0258832. doi: 10.1371/journal.pone.0258832 (PMC8759659; doi:10.1371/journal.pone.0258832)
Supplement: Attachment — Submitted filename: Response to Reviewers.docx [file pone.0258832.s002.docx]

# RESPONSE TO REVIEWERS

Dear Dr Steinborn,

Once more we’d like to thank you and the reviewers for the time and further comments concerning our paper “Rapid detection of social interactions is the result of domain general attentional processes”. We appreciate the opportunity to clarify and re-submit the work after. In the following we describe our response to the reviewer feedback. Reviewer comments are shown in black and our responses are shown in blue.

Yours sincerely,

Jonathan Flavell, Harriet Over, Tim Vestner, Richard Cook, Steven Tipper

-o0o-

## REVIEWER #1

I feel that the authors have addressed all of my comments adequately and I strongly endorse publication.

Thank you for your time and for your recommendation.

## REVIEWER #3

The authors have addressed most of my earlier comments; I have a couple additional suggestions, but in general I believe with their revisions (along with their responses to other reviewers' comments), the manuscript is now in a better shape.

Thank you for another thorough and considered review once again.

Regarding my point about labeling vs. perceiving social interactions, I see that the authors edited the example I provided, and I appreciated that the new introductory paragraph was worded accordingly. I disagree however that Experiment 2 rules out possible contributions of demand characteristics, since it looks like the instructions and question still mentioned “characters” that were “facing towards each other” – which is of course less apparent as a manipulation compared to speech bubbles, but could still plausibly be perceived as such.

To clarify, in our previous Response to Reviewers we “…the question of front/face may not be as susceptible to demands…” rather than stating ruling them out entirely (which the present set of studies cannot do conclusively).

Note that we have added some additional text to *Experiment 2 > Results and Discussion*.

In the previous round of review, I had discussed the results of Expt. 5, namely that seagulls facing each other are prioritized in visual search (and how it is consistent with a ‘social’ interpretation: if during the search task participants automatically perceive the stimuli as seagulls – regardless of which prime they had been exposed to – then the advantage for inward seagulls might reflect a prioritization of ‘facing’ vs. ‘non-facing’ dyads). The authors rule out this interpretation mentioning that (1) condition assignment was randomized between subjects, and (2) “there was no effect of seagulls”. For (1), I am not sure how random assignment might speak to the possibility that all participants might perceive the stimuli as seagulls during the search task, regardless of previous priming; and (2) also confused me, since my understanding is there was no effect of prime, but there was an effect of stimulus orientation for seagulls (faster for facing vs. non-facing seagulls, i.e. faster for non-facing vs. facing rabbits). I think this might just be a misunderstanding, but if not (i.e. if there was instead no effect of facing seagulls), the results section for Expt. 5 should be clarified.

Re. (1). Perhaps we had crossed wires. We thought that your description of seagulls as “prioritized in Expt. 5” was implying a methodological prioritisation.

Re. (2). You are correct in that RTs were affected by seagull orientation but not by prime type. We cannot find the quote “there was no effect of seagulls” in our previous Manuscript or Response to Reviewers. Assuming you meant our words “There was no priority for seagulls” in Response to Reviewers, this is explained by the above point.

We have edited the text in *Experiment 5 > Results and Discussion* and hope that this clarifies our own view on these data.

I might have missed this, but I don’t think the stimulus generation method for the ‘round-face’ videos has been clarified in the manuscript?

We did not realise that you were requesting an edit to the manuscript in your previous comment. We have now added the following to *Experiment 2 > Method > Task blocks and social priming*: “The pointed and rounded videos were identical apart from the orientation of the teardrop which was mirrored to create a pointed or rounded prime.”

I still see that the conditions of Expt. 7 labeled as ‘rabbit’ and ‘orange’ in the RT file, the MT file, and the data exclusion file.

Thankyou for identifying this error. These documents have now been corrected.

The averages for Expt. 2 in the raw data file seem to be inconsistent with the figure (i.e. mean RT for inward point = 1014ms, inward round = 1003ms, but the depicted mean in Fig. 10 is higher for round). (As an aside, one would naturally associate pointed vs. rounded shapes with the point vs. round condition, but pointed vs. rounded shapes actually signal SOA in the figure, while point vs. round condition is signaled by color.)

The figure has been corrected. The data are corrected but the figure labels were switched accidentally. We have corrected this and taken the opportunity to switch the icons in Figure 10 so that circles are for the rounded front condition and the triangles are for the pointed front condition.

The OSF stimuli folder for Exp. 6 contains priming videos and search targets – but wasn’t Exp. 6 simply a cueing task?

You are correct. There were no priming videos in Experiment 6 and these were uploaded in error. The six files have been removed from the OSF.

Apologies if I missed this in the manuscript, but it should be clarified that RT was computed as the time of spacebar release (i.e. without the reaching movement).

Thankyou for identifying this omission. We have added the following text to *Experiment 2 > Method > Practice & trial composition*: “Reaction times were measured from the moment the four simultaneously presented pairs appeared to the moment of space bar release. Movement time is measured from the moment of space bar release to the moment of screen contact.”

Both the main text and supplement state that in Expt. 1, 33 participants were tested and 26 remained following exclusions; but of the (presumably 7) excluded subjects, only 6 are mentioned (2 based on accuracy, and 4 based on RT).

We have double checked and it was the recruited size that was in error. It has been corrected to “Thirty-three two participants were tested…”. Thank you for identifying this mistake.
